# Low back pain in beta thalassemia major revealing sacral extramedullay hematopoeisis: A case report

**DOI:** 10.1002/ccr3.4258

**Published:** 2021-05-24

**Authors:** Rita W. Ahmad, Lina A. Okar, Abdelhaleem Elhiday, Hussam Almasri, Fateen Ata, Elsayed Ahmed Mounir, Ali Barah, Mohamed Abdelrazek, Amna Gamil, Mouhammad Z. Sharaf Eldean, Mohamed A. Yassin

**Affiliations:** ^1^ Department of Family Medicine Hamad Medical Corporation Doha Qatar; ^2^ Department of Internal Medicine Hamad General Hospital Hamad Medical Corporation Doha Qatar; ^3^ Department of Orthopedic Surgery Hamad Medical Corporation Doha Qatar; ^4^ Department of Radiology Hamad Medical Corporation Doha Qatar; ^5^ Hematology Oncology Department National Centre for Cancer Care & Research Doha Qatar; ^6^ Department of Pathology Hamad General Hospital Doha Qatar

**Keywords:** beta thalassemia, extramedullary hematopoiesis, low back pain, thalassemia

## Abstract

Extramedullary hematopoiesis (EMH) is a well‐known complication of beta thalassemia major and frequently occurs in typical sites such as liver or spleen. However, when presenting in unusual sites as sacrum, other diagnosis should be excluded by histopathology prior to deciding on treatment plan.

## Graphical abstract

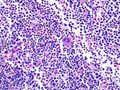

## 1 INTRODUCTION

Extramedullary hematopoiesis (EMH) represents the production of blood cells outside of the bone marrow and occurs in a variety of hematologic illnesses, primarily beta thalassemia major. Although EMH usually occurs in the liver, spleen, and lymph nodes, it may also occur in the spinal canal or sacral bone leading to spinal cord compression (SCC). This case describes SCC as a consequence of EMH presenting as a mass in presacral area.

Beta thalassemia major is the severe form of beta thalassemia, which is caused by mutations in beta globin gene, either reduced (β^+^) or absent (β^0^) in hemoglobin A resulting in unbound α globin chains that accumulate in erythroid precursors in the bone marrow and in the mature erythrocytes leading to ineffective erythropoiesis and peripheral hemolysis. About 1.5% of the world population are carriers of β thalassemia.^1^

In severe disease, extramedullary hematopoiesis (EMH)‐which is production of blood elements outside the bone marrow‐occurs mostly in the liver and the spleen but in rare cases can manifest as bony masses that behave clinically like tumors, leading to optic nerve atrophy, spinal cord compression, and other clinical scenarios.^2^, ^3^

Here, we present a case of a 31‐year‐old Pakistani woman with transfusion‐dependent beta thalassemia (TDBT), who complained of low back pain due to a mass reflecting extramedullary hematopoiesis which very rarely occurs in the sacrum as there was only one case reported in the literature and this is the second one.

## 2 CASE PRESENTATION

A 31‐year‐old Pakistani female presented with generalized body pain and low back pain. Her past medical history was remarkable for transfusion‐dependent beta thalassemia (TDBT) major since childhood complicated with iron overload as per her cardia and hepatic magnetic resonance imaging (MRI) reports, receiving iron chelation therapy (Deferasirox 1080 mg daily), type II diabetes mellitus on insulin therapy and migraine. Her past surgical history includes splenectomy on 2012. She has a long history of body pain. She was recently admitted due to COVID‐19 infection and discharged from quarantine facility 1 week earlier after a smooth course of infection.

She presented to the emergency department complaining of body pain for the last few days and dysuria. She has similar complaints on and off for many years, yet the pain increased recently, and it is mainly in the hip and lower abdomen area (suprapubic), the pain increases with urination, she has also back pain for the last 3 months more severe at night, responds partially to analgesia, radiates to the hip, and is limiting her movement. On admission, vitals were given as:

BP 107/67 mm Hg,
HR 95/min
Temperature 36.7^°^
Saturation 96% on room air.

Primary investigations included: Labs, MRI pelvis and hips, Abdomen ultrasound (US) showed the following:

Labs: On admission (Table 1)

**TABLE 1:** Lab results

Lab Test	Result	Reference range
WBCs	20.4	(4‐10 x 10^3^/UL)
RBCs	3.6	(4.5‐5.5 x 10^6^)
Platelets	462	(150‐400 × 10^3^/UL)
Hemoglobin	9.8	(13‐17 gm/dL)
Hematocrit	30.3	(36%‐46%)
MCV	83.5	(83‐101 fL)
ANC	9.8	(2‐7 x 10^3^/UL)
Lymphocyte count	8.4	(1‐3 x 10^3^/UL)
Na	133	(136‐145 mmol/L)
Total Bilirubin	25	(0‐21 Umol/L)
Albumin	29	(35‐50 gm/L)
ALP	227	(35‐104 U/L)
ALT	169	(0‐33 U/L)
AST	134	(0‐32 U/L)
Iron	29	(2.9‐22.9 Umol/L)
TIBC	25	(45‐80 Umol/L)
Fe saturation	116	(15%‐45%)
HbA1c	10.6%	(4%‐5.6%)
CRP	7.4	(0‐5 Umg/L)
Ferritin	19, 786	(8‐252 mcg/L)
Hepatitis panel	negative	
TSH	20.7	(0.5‐4.30 mIU/L)
FT4	15.2	(12.9‐20.6 pmol/L)

Her labs revealed hypothyroidism, so she was started on Levothyroxine therapy.

Xray pelvis and left hip

Showed decreased bone density, bilateral coxa profunda.
US abdomen showed mildly enlarged fatty liver (18.2 cm).

US pelvic: Normal study.

MRI Hips and pelvic: Diffuse bone marrow reconversion and Presacral soft tissue mass as described, most likely extramedullary hematopoiesis.

So as per MRI, the patient has extramedullary hematopoiesis presenting as soft tissue mass in the presacral area, from surgical point of view, the mass is not accessible, but still need to be excised. After that cervical and lumbar MRI spine was done to look if there are other extramodular hematopoietic sites and it showed:

Diffusely decreased T1 marrow signal intensity of the entire visualized bones which is in keeping with red marrow reconversion. Redemonstrations of a presacral well‐defined lobulated soft tissue mass measuring 5.3 × 3 cm, it is separated from the underlying sacrum, no sacral destruction. The mass is showing mild postcontrast enhancement suggestive of extramedullary hematopoiesis. Another similar smaller extradural mass measuring 2 cm is seen posterior to S1 vertebral body at the midline laterally displacing S1 traversing nerve roots.

**FIGURE 1:**
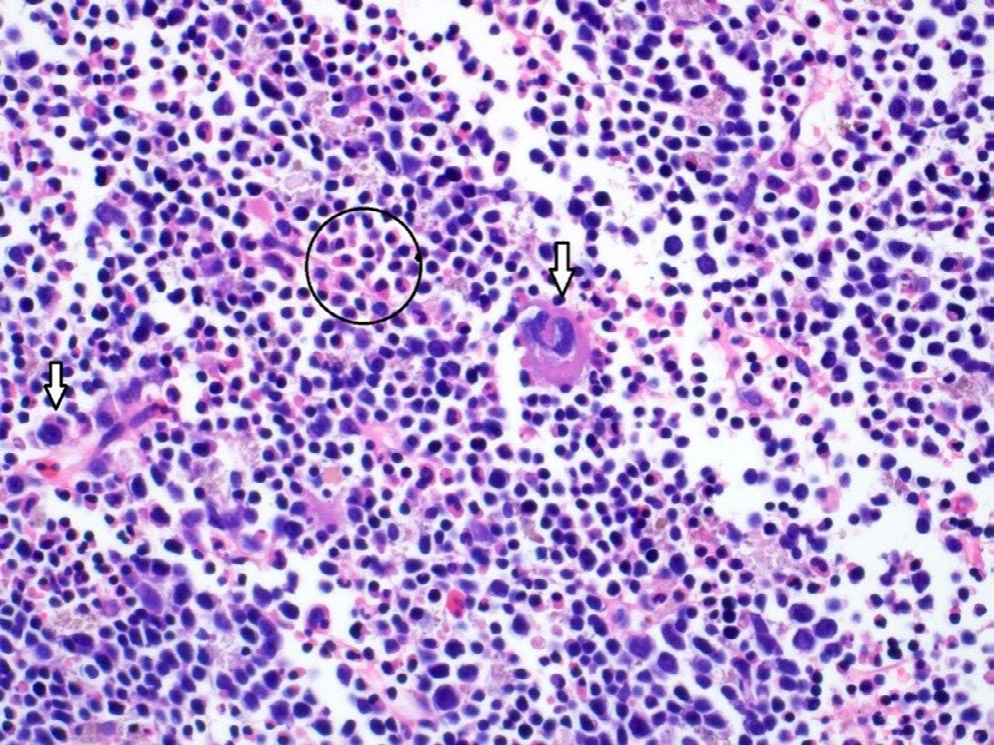
High magnification image of H&E stained slide showing lymphoid tissue composed of small lymphocytes with scattered megakaryocytes (arrows) and clusters of myeloid and erythroid cells (circled)

**FIGURE 2:**
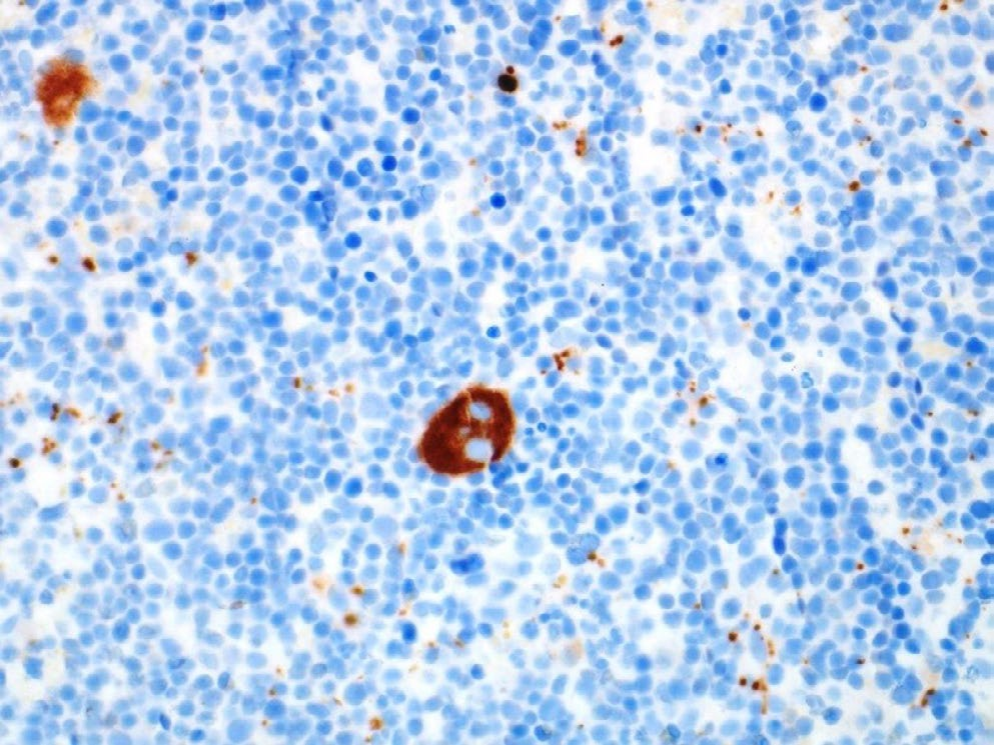
Immunohistochemical stain “CD61”, highlighting the cytoplasm of megakaryocyte in brown chromogen and confirming the lineage

**FIGURE 3:**
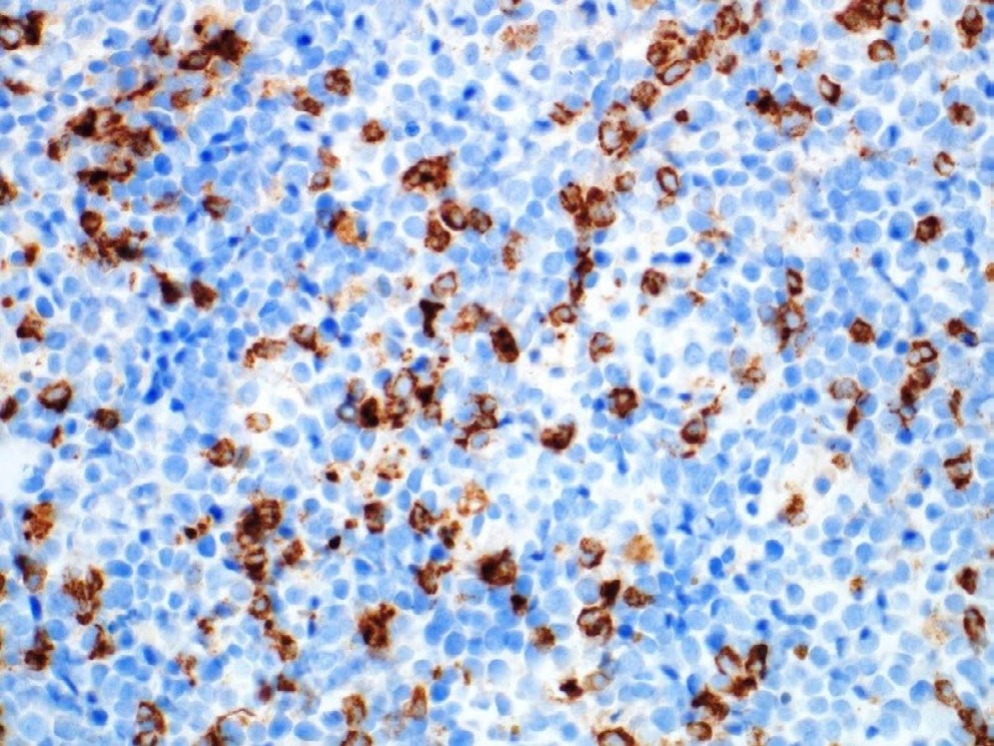
Immunohistochemical stain “MPO”, highlighting the myeloid precursors

**FIGURE 4:**
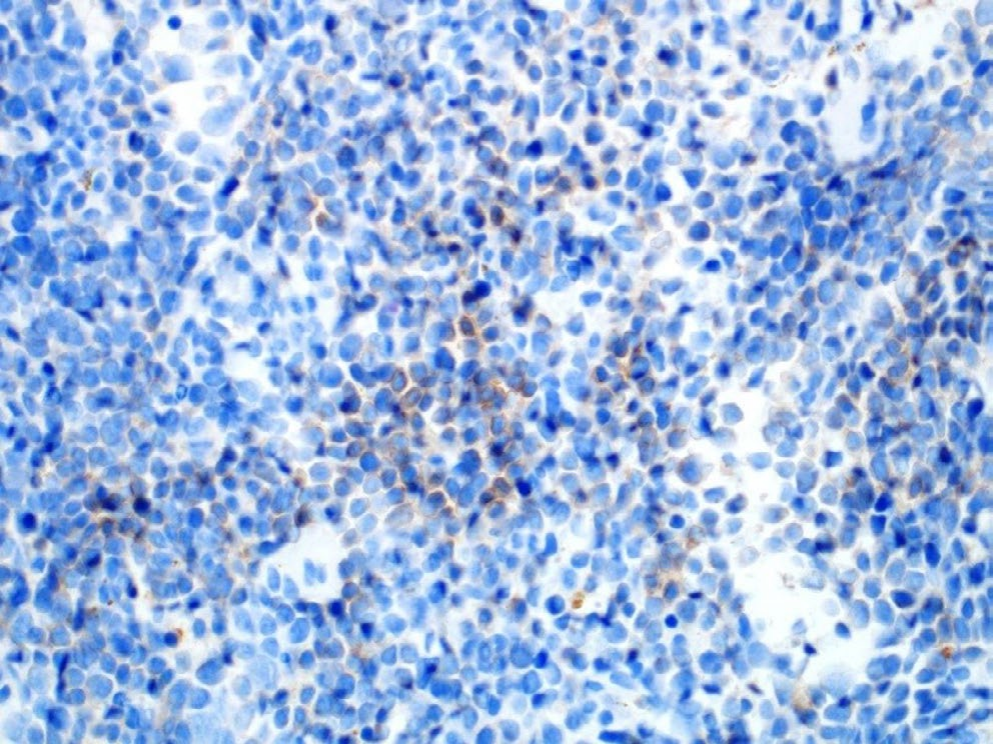
Immunohistochemical stain “Hemoglobin A”, highlighting the erythroid precursors

***To further establish the diagnosis, a bisopsy has been taken from the mass which demostrated the extramedullary hematopoeisis. (Figures 1,2,3,4)***

​

Currently, she is still on blood transfusion to keep HB more than 10 in case of any surgical procedure was decided to be done, plan now is toward the excision of the mass. The patient is taking the following medications: Celecoxib 200 mg PRN, deferasirox 1080 mg oral daily, insulin therapy and vitamin D supplementation, Levothyroxine 75 mg daily, and tramadol 50 mg oral BID.

**FIGURE 5:**
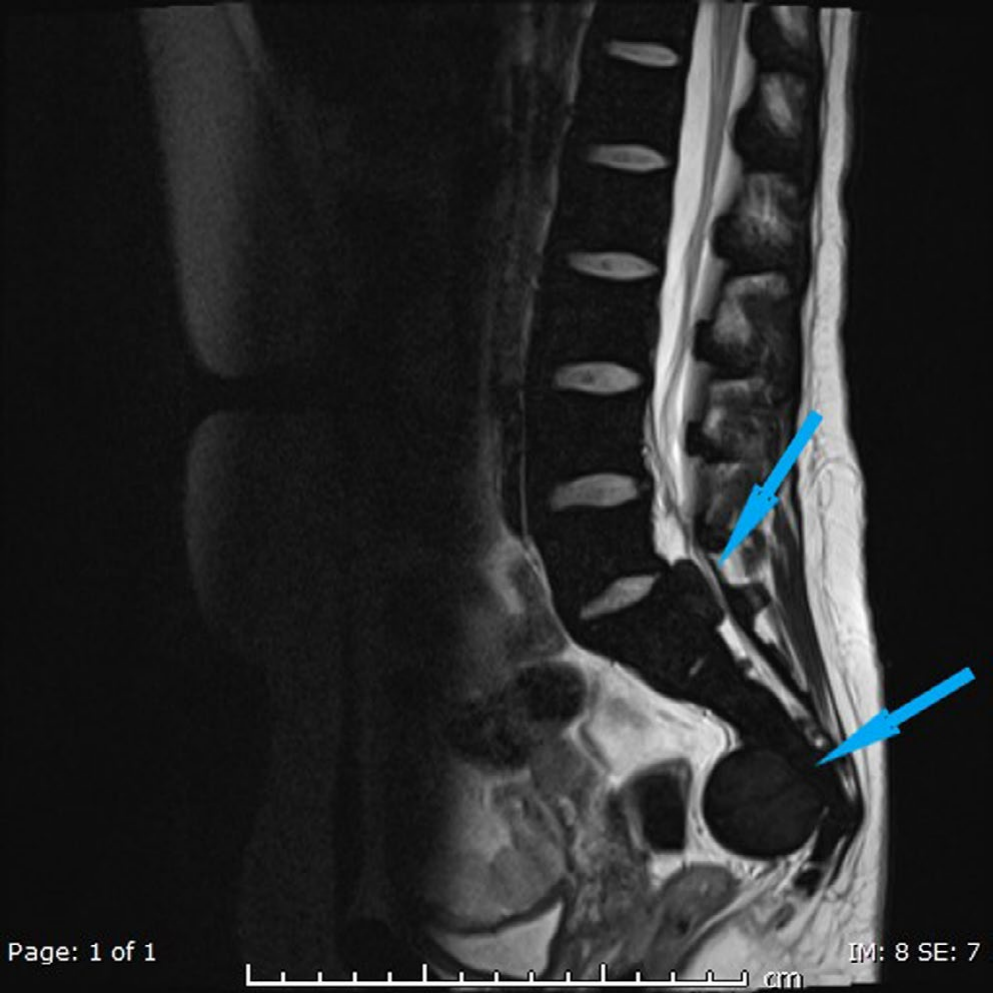
Sagittal T2 WI of the LSS shows diffuse low‐signal intensity of the bone marrow of LSS (known case of thalassemia major) with well‐defined mass of the same signal intensity (5.3 x 3 cm) in presacral region (blue arrow) and another similar smaller mass (2 × 1 cm) in anterior extradural space posterior to S1 level (Blue arrow)

**FIGURE 6:**
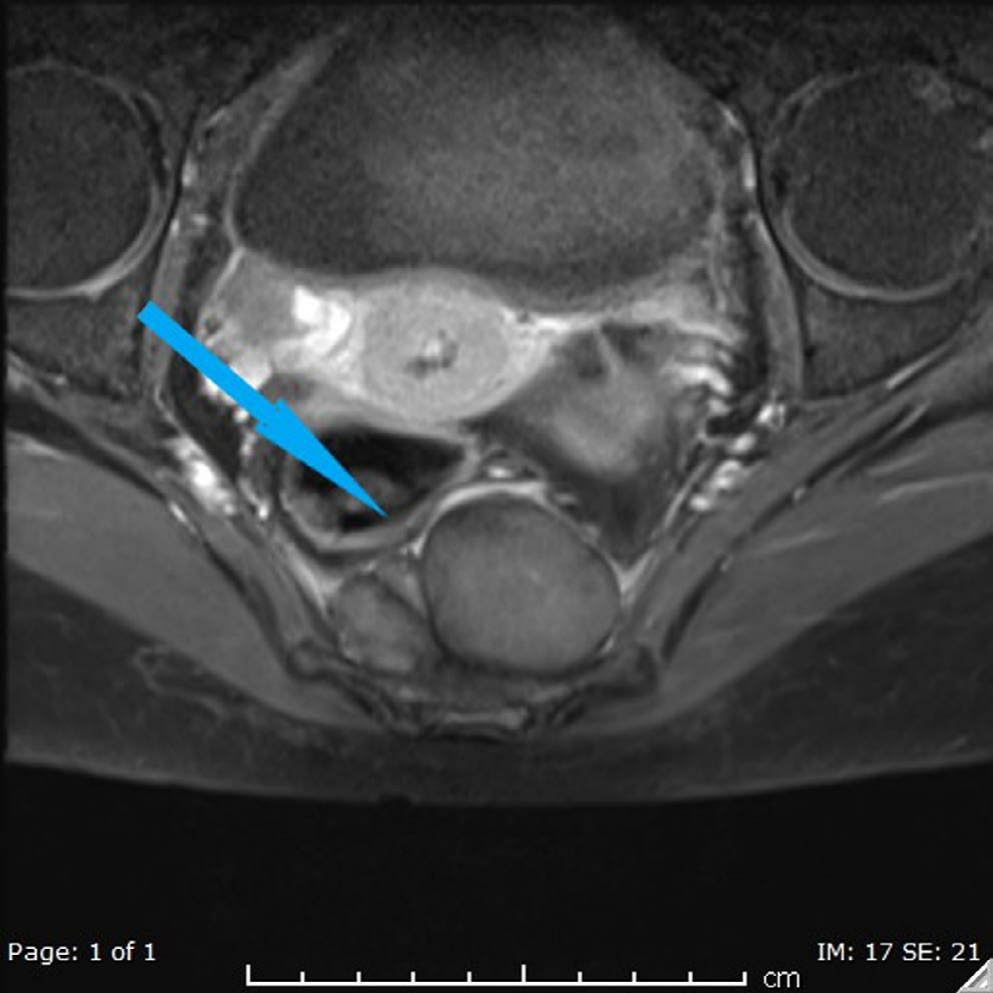
Axial postcontrast fat saturated image at presacral region shows mild enhancement of the presacral mass (Blue arrow). No definite infiltration of the sacrum

**FIGURE 7:**
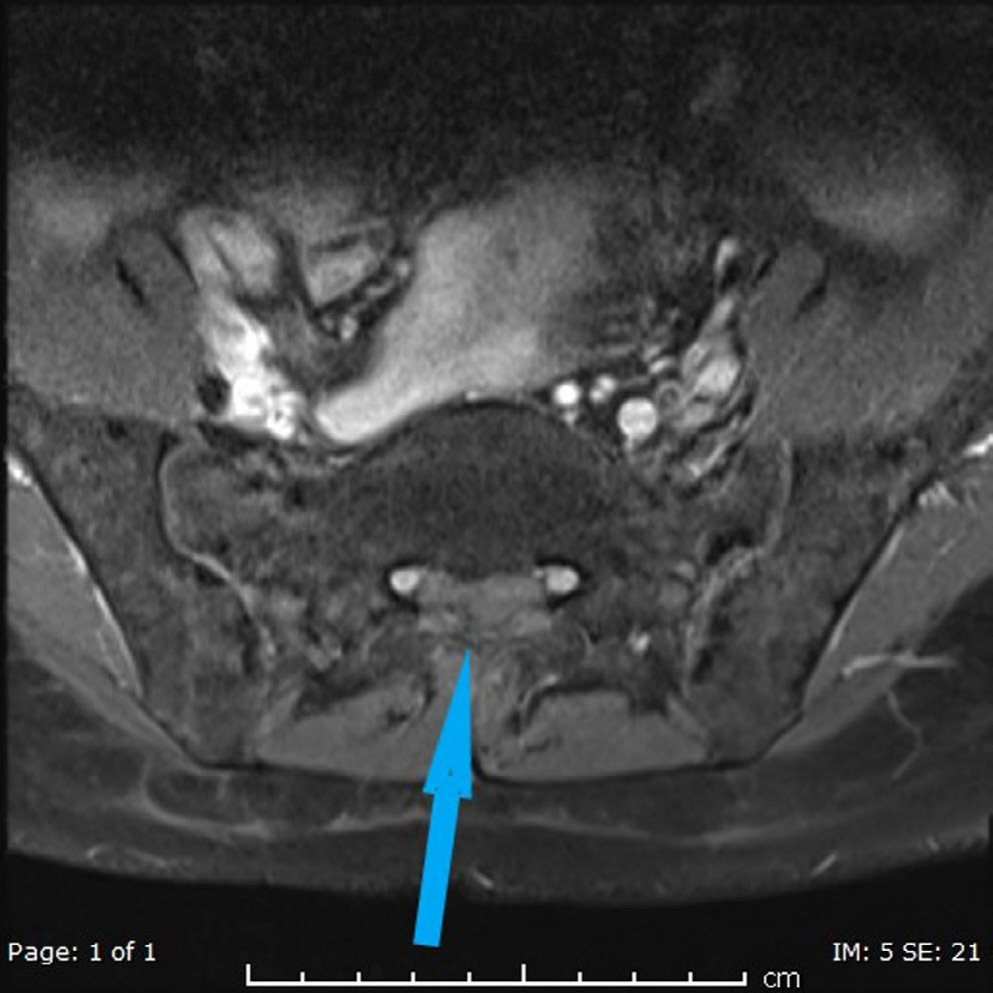
Axial postcontrast fat saturated image at S1 level shows mild enhancement of anterior extradural mass (blue arrow) at the midline laterally displacing S1 traversing nerve roots

The following images show MRI lesions and biopsy results (Figures 1, 2, 3, 4 and Figures 5, 6, 7).

## 3 DISCUSSION

Thalassemia is the most common hemoglobinopathy, almost 5% worldwide have at least one thalassemia defect allele. It is highly prevalent in Southeast Asia, the Asian‐Indian subcontinent, and the Mediterranean region.^1^, ^4^

It is categorized into major two groups, α thalassemia and β thalassemia.

α thalassemia is caused by deletions in one or more of the four alpha globin genes. Deletion of two alpha genes causes thalassemia alpha minor (–, αα) or (‐α,‐α), while loss of three genes results in hemoglobin H disease (–, ‐α), and deletion of all four genes leads to hemoglobin Barts or hydrops fetalis.

β thalassemia on the other hand, results from mutations rather than deletions in beta globin genes ranging from reduced production of beta chains (β^+^) and asymptomatic disease as in beta thalassemia trait or minor to absence of beta chains causing severe course of the disease seen in beta thalassemia major (β^0^).^4^ The absence of beta globin chains results in excess unbound α globin chains that accumulate and precipitate in erythroid precursors in the bone marrow and in the mature erythrocytes, leading to ineffective erythropoiesis and peripheral hemolysis.^1^

Transfusion‐dependent beta thalassemia or also known as Cooley's anemia refers to the most severe form of the disease where there is minimal to no beta globin chain production and as a result little to absent HbA.

Patients with beta thalassemia major have profound and lifelong transfusion‐dependent anemia, which affects their quality of life and performance status, especially among children patients as it largely affects their academic performance as well as their emotional and psychological status.^5^ Symptoms typically begin to manifest relatively late after birth (6‐12 months of age) because major hemoglobin in newborns is fetal hemoglobin (HbF), which consists of gamma chains instead of beta chains. Symptoms include anemia which is severe and profound and if untreated can be as low as 3‐4 mg/dL, it is typically microcytic hypochromic with elevated RBC count. Gallstones formation and jaundice due to chromic hemolysis which also leads to hepatosplenomegaly which can be exacerbated by extramedullary hematopoiesis. Patients who develop cytopenias and shortened survival of transfused RBCs may be evaluated for splenectomy.^6^ TDBT as the name implies carries the risks associated with recurrent blood transfusions, especially iron overload that requires monitoring and management by long‐term iron chelation therapy to prevent iron overload complications such as cardiomyopathy and endocrinopathy, which carry high morbidity and mortality rates.^7^ However, iron overload is a one complication that paves the way for subsequent serious complications. Osteoporosis is recognized due to different etiologies including chronic anemia, ineffective hematopoiesis and bone marrow expansion, calcium and zinc deficiencies. But the exact mechanism is still not fully understood.^8^ Other complications include thyroid disease particularly hypothyroidism, primary rather than secondary,^9^ as seen in our patient with TSH of 20 mIU/L with no other signs or symptoms suggesting secondary cause, and was started on levothyroxine during admission. Excess iron also leads to diabetes and glucose abnormalities, hypogonadotropic hypogonadism due to chronic liver disease and impaired pituitary axis which also can cause growth hormone deficiency.^10^, ^11^, ^12^

Extramedullary hematopoiesis is the production of blood cells outside the bone marrow that typically occurs in liver and spleen and leads to hepatosplenomegaly, although rarely can result in skeletal malformations such as facial deformities and change in body habitus. It also may manifest in the mediastinum and produce symptoms as cough or shortness of breath mimicking thoracic tumors.^2^ However, spinal cord compression (SCC) is a rare manifestation of EMH although it may be asymptomatic, EMH‐associated SCC can lead to permanent neurological injury, and when that occurs, surgical decompression should be considered with or without radiotherapy, although surgery is not always possible and may carry potential complications.^13^

Magnetic resonance imaging is the diagnostic modality of choice, and biopsy is not always needed to confirm the diagnosis, but when performed it can be CT‐guided as was done in our case.^13^

Sheikh et al^14^ described 16 cases of EMH diagnosed by ultrasound‐guided fine needle aspiration (FNA) which can be less invasive than core biopsy.

In our patient, the main symptom was low back and low abdominal pain, without neurological symptoms. The MRI showed soft tissue mass in the presacral area representing EMH. Patient was started on hydroxyurea and hyper transfusion. Treatment options for EMH at this site are either radiotherapy or surgical excision. Because of the patient's young age and the risk of infertility, surgical excision was preferred to radiotherapy particularly as the lesion is also well‐localized and operable.

## 4 CONCLUSION

Extramedullary hematopoiesis is a well‐established complication of thalassemia major and can manifest in variable forms ranging from asymptomatic hepatosplenomegaly to skeletal malformations causing serious adverse effects such as SCC which is rarely encountered but should always be held in mind when assessing patients with TDBT.

## CONFLICT OF INTEREST

None declared.

## AUTHOR CONTRIBUTIONS

RWA: involved in first author, manuscript writing, and literature review. LAO: involved in case‐presentation writing. HA: involved in manuscript writing. FA: involved in literature review. AE: involved in literature review. AG: involved in literature review. AB: involved in providing radiological images. MZSE: involved in histopathlogic diagnosis and slides. EAM: involved in surgical point of view and literature review. MAY: involved in mentorship, manuscript writing, and literature review.

## ETHICAL APPROVAL

Ethical Approval was obtained by Medical Research Center (MRC) under ID MRC‐04‐20‐883 on October 13th, 2020. The article processing charges were funded by Qatar National Library (QNL).

## CONSENT STATMENT

Published with written consent of the patient.

## Data Availability

All data generated during this study are included in this article.

## References

[1] De Sanctis V , Kattamis C , Canatan D , et al. β‐thalassemia distribution in the old world: an ancient disease seen from a historical standpoint. Mediterr J Hematol Infect Dis. 2017;9(1):e2017018. 28293406 10.4084/MJHID.2017.018 PMC5333734

[2] Abdulla MA , Yassin MA , Abdelrazek M , et al. A persistent cough as atypical clinical presentation of intrathoracic extramedullary hematopoiesis (EMH) in a female with thalassemia intermedia. Acta Biomed. 2018;89(Suppl 2):41. 10.23750/abm.v89i2-S.7086 PMC6179037 29451228

[3] Garg K , Singh PK , Singh M , Chandra PS , Sharma BS . Long segment spinal epidural extramedullary hematopoiesis. Surg Neurol Int. 2013;4:161. 10.4103/2152-7806.123657 24404404 PMC3883269

[4] Khan AM , Al‐Sulaiti AM , Younes S , Yassin M , Zayed H . The spectrum of beta‐thalassemia mutations in the 22 Arab countries: a systematic review. Expert Review of Hematology. 2021 Jan 2. 10.1080/17474086.2021.1860003 33317346

[5] Nashwan AJ , Yassin MA , Babu GD , et al. Quality of life among adolescents aged 14 to 18 years with beta‐thalassemia major (TM) in Qatar. Acta Biomed. 2018;89(Suppl 2):16. 29451225 10.23750/abm.v89i2-S.7083 PMC6179034

[6] Tassiopoulos T , Rombos Y , Konstantopoulos K , Revenas K , Tassiopoulos S , Aessopos A . Spleen size in beta‐thalassaemia heterozygotes. Haematologia (Budap). 1995;26(4):205‐209. 7590515

[7] Kanbour I , Chandra P , Soliman A , et al. Severe liver iron concentrations (LIC) in 24 patients with β‐thalassemia major: correlations with serum ferritin, liver enzymes and endocrine complications. Mediterr J Hematol Infect Dis. 2018;10(1):e2018062. 30416694 10.4084/MJHID.2018.062 PMC6223579

[8] Yassin MA , Soliman AT , De Sanctis V , Abdelrahman MO , Bedair EM , AbdelGawad M . Effects of the anti‐receptor activator of nuclear factor kappa B ligand denusomab on beta thalassemia major‐induced osteoporosis. Indian J Endocrinol Metab. 2014;18(4):546. 25143915 10.4103/2230-8210.137516 PMC4138914

[9] Soliman AT , Al Yafei F , Al‐Naimi L , et al. Longitudinal study on thyroid function in patients with thalassemia major: high incidence of central hypothyroidism by 18 years. Indian J Endocrinol Metab. 2013;17(6):1090. 24381890 10.4103/2230-8210.122635 PMC3872691

[10] De Sanctis V , Soliman AT , Elsedfy H , et al. Diabetes and glucose metabolism in thalassemia major: an update. Expert Rev Hematol. 2016;9(4):401‐408. 26697756 10.1586/17474086.2016.1136209

[11] De Sanctis V , Soliman AT , Candini G , et al. Insulin‐like growth factor‐1 (IGF‐1): demographic, clinical and laboratory data in 120 consecutive adult patients with thalassaemia major. Mediterr J Hematol Infect Dis. 2014;6(1):e2014074. 25408860 10.4084/MJHID.2014.074 PMC4235482

[12] De Sanctis V , Elsedfy H , Soliman AT , et al. Acquired hypogonadotropic hypogonadism (AHH) in thalassaemia major patients: an underdiagnosed condition? Mediterr J Hematol Infect Dis. 2016;8(1):2016001. 10.4084/MJHID.2016.001 PMC4696472 26740862

[13] Cos E , Keskin A , Süzer T , Sermez Y , Kildaci T , Tahta K . Spinal cord compression secondary to extramedullary hematopoiesis in thalassemia intermedia. Eur Spine J. 1998;7(6):501‐504. 10.1007/s005860050114 9883960 PMC3611303

[14] Sheikh U , Rodic N , Maleki Z . Extramedullary hematopoiesis: cytomorphologic, histologic, and radiologic findings in sixteen cases. Acta Cytol. 2015;59(2):144‐148. 10.1159/000376602 25871506

